# Development and Proof of Concept of a Compact Metallic Reactor for MIEC Ceramic Membranes

**DOI:** 10.3390/membranes11070541

**Published:** 2021-07-16

**Authors:** Sonia Escolástico, Falk Schulze-Küppers, Stefan Baumann, Katja Haas-Santo, Roland Dittmeyer

**Affiliations:** 1 IMVT, Karlsruhe Institute of Technology, 76344 Eggenstein-Leopoldshafen, Germany; katja.haas-santo@kit.edu (K.H.-S.); roland.dittmeyer@kit.edu (R.D.); 2 Institute of Energy and Climate—IEK1 Materials Synthesis and Processing, Forschungszentrum Jülich GmbH, 52425 Jülich, Germany; f.schulze@fz-juelich.de (F.S.-K.); s.baumann@fz-juelich.de (S.B.)

**Keywords:** metallic compact reactors, MIEC membranes, catalytic membrane reactors, O_2_ separation

## Abstract

The integration of mixed ionic–electronic conducting separation membranes in catalytic membrane reactors can yield more environmentally safe and economically efficient processes. Concentration polarization effects are observed in these types of membranes when O_2_ permeating fluxes are significantly high. These undesired effects can be overcome by the development of new membrane reactors where mass transport and heat transfer are enhanced by adopting state-of-the-art microfabrication. In addition, careful control over the fluid dynamics regime by employing compact metallic reactors equipped with microchannels could allow the rapid extraction of the products, minimizing undesired secondary reactions. Moreover, a high membrane surface area to catalyst volume ratio can be achieved. In this work, a compact metallic reactor was developed for the integration of mixed ionic–electronic conducting ceramic membranes. An asymmetric all-La_0.6_Sr_0.4_Co_0.2_Fe_0.8_O_3–δ_ membrane was sealed to the metallic reactor by the reactive air brazing technique. O_2_ permeation was evaluated as a proof of concept, and the influence of different parameters, such as temperature, sweep gas flow rates and oxygen partial pressure in the feed gas, were evaluated.

## 1. Introduction

Process intensification aims to increase the production capacity, to decrease the energy consumption and to reduce waste with the subsequent reduction of the production costs. The development of new processes and equipment is a key factor to reach these goals. In this context, the integration of catalytic membrane reactors employing mixed ionic–electronic conductors (MIEC) based separation membranes could yield more environmentally safe and economically efficient processes. These membrane reactors allow for the controlled removal or feeding of O_2_, and consequently, enable to surpass equilibrium conversion or increase product selectivity in reactions, such as oxidative dehydrogenation of hydrocarbons, oxidative coupling of methane or partial oxidation of methane [1,2,3,4,5,6,7,8]. MIEC materials allow the transport of oxygen ions and electrons through their crystal structure. The O_2_ separation is then driven by the O_2_ partial pressure gradient across the membrane. Some of the needed targets for these types of membranes are listed: (a) high permeation fluxes; (b) low cost and (c) stability and durability.

Amongst the different MIEC materials, perovskites (ABO_3–δ_) and fluorites (AO_2_ where A is a cation such as Zr^4+^ or Ce^4+^) based compounds are the most promising as oxygen permeable membranes [9,10]. The most studied perovskites are based on Sr(Co,Fe)O_3–δ_ (SCFO) and Ba_0.5_Sr_0.5_Co_0.8_Fe_0.2_O_3–δ_ (BSCF) materials. In order to improve their O_2_ permeation and stability, they can be tailored by substituting the metal cations [11,12,13,14]. In addition, O_2_ transport can also be improved by decreasing the membrane thickness, giving rise to important oxygen permeation fluxes [15,16,17]. These thin membranes are normally deposited on a porous substrate for ensuring the mechanical stability. This support results in an additional mass transport resistance, giving rise to a reduction of the expected O_2_ flux because of a decrease in the O_2_ partial pressure difference over the actual membrane. This limitation has been partially overcome by using supports with engineered porosity [18,19,20]. The highest O_2_ flows by using MIEC-based membranes have been achieved with thin membranes made of BSCF with a thickness of around 30 μm [15]. Lower but important O_2_ fluxes and improved stability were obtained for LSCF (La_0.6_Sr_0.4_Co_0.2_Fe_0.8_O_3–δ_) thin film membranes [15]. Another possible limitation is a slow catalytic surface exchange that becomes the limiting step for O_2_ permeation at temperatures below 700 °C. This can be overcome by the deposition of porous activation layers on the membrane faces [3,21]. However, concentration polarization can also occur when O_2_ permeating fluxes are high due to the gas phase mass transport resistance. This causes an increase in the oxygen concentration on the membrane surface in the permeate side, which subsequently decreases the driving force. This last drawback could be alleviated by the development of new membrane reactors where mass transport and heat transfer are enhanced by adopting state-of-the-art microfabrication. In addition, careful control over the fluid dynamics regime in the reactor could also allow for a more rapid extraction of the products in a catalytic membrane reactor, minimizing secondary reactions [22]. The integration of microchannels in these metallic compact reactors provides a high membrane surface area to catalyst volume ratio [23].

On the other hand, tested ceramic membranes are mostly placed in ceramic or quartz housings, which are prone to damage because they are brittle and breakable. The use of compact metallic reactors could help to overcome the abovementioned drawbacks and to enable high-pressure operation. Furthermore, this membrane reactor concept could enable an easier use and scale-up of ceramic membranes in a stable housing [24]. One of the major challenges of the integration of ceramic membranes in metallic reactors is the sealing of the membrane to the metal parts due to the difference of the thermal expansion coefficients. Brazing techniques seem to be the most promising option to get a high temperature sealing of ceramic and metals [25]. Several successful attempts have been obtained in the last years by using Ag-CuO based pastes, allowing for a robust sealing [21,22].

This study is based on the manufacturing and testing of a metallic membrane module for the integration of ceramic membranes. This work is divided into several steps: (a) development of a compact metallic membrane reactor that allows it to work at high pressure and high flow velocity; (b) development of the sealing process by a brazing technique; and (c) O_2_ permeation measurements with an asymmetric all-La_0.6_Sr_0.4_Co_0.2_Fe_0.8_O_3–δ_ (LSCF) membrane as a proof of concept.

## 2. Materials and Methods

### 2.1. Membrane Reactor

**Figure 1:**
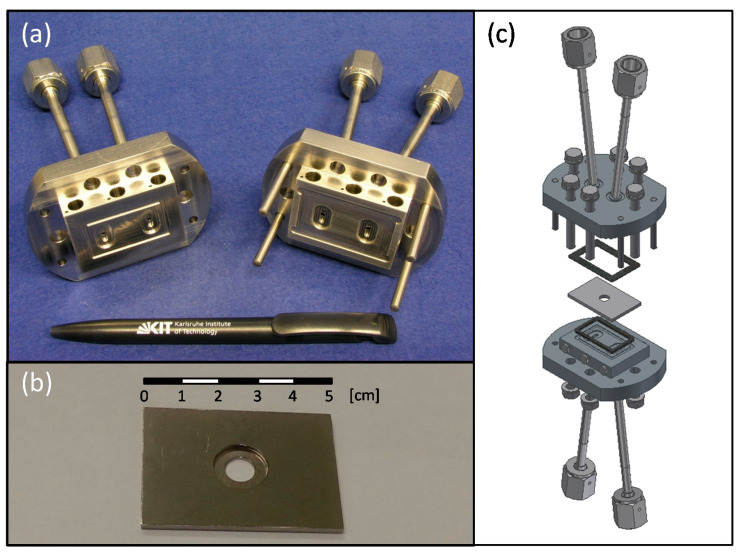
Housing (**a**), membrane module (**b**) and schematic of the membrane reactor (**c**).

**Figure 2:**
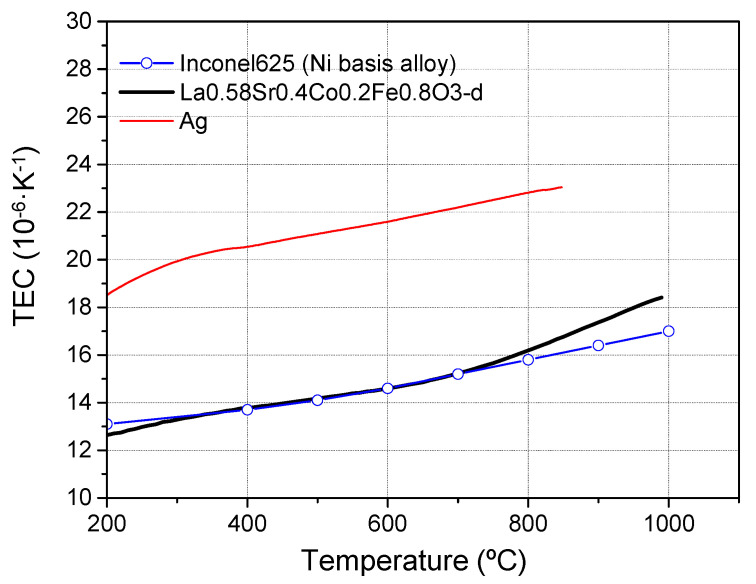
TEC of LSCF, Ag and Inconel 625 alloy [27,30,31,32].

A compact metallic membrane reactor was designed to integrate ceramic oxygen permeable membranes (Figure 1). The reactor consisted of a housing with two separated parts that provided inlets and outlets for sweep gas (permeating chamber) and feed gas (feed chamber) (Figure 1a). The housing was made of high temperature resistant metal alloy (Nicrofer^®^ 3220H/Alloy 800 (1.4876), ThyssenKrupp, Essen, Germany). The membrane module consisted of a rectangular plate made of Inconel alloy 625 with a disk holder as shown in Figure 1b. The module separated both chambers of the reactor (sweep and feed) and allocated the membrane. Inconel alloy 625 was selected due to the similar thermal expansion coefficient (TEC) compared to La_0.6_Sr_0.4_Co_0.2_Fe_0.8_O_3–δ_ (LSCF) as shown in Figure 2 where TEC of LSCF, Inconel 625 alloy and Ag (used in the brazing) are plotted. The disk holder used in this work had an external diameter of 13 mm and an internal diameter of 7 mm, which was the effective area of the membrane. In addition, modules with different holder geometry were also developed. The membrane module was leak-tight integrated in the housing by using phlogopite mica sealings, which can be observed in the schematic of the membrane reactor.

The membrane reactor worked in two different configurations: (a) co-current: where inlets of the sweep and feed sides were located in the same extreme of the reactor and (b) counter-current: inlets were in opposite extremes. These two different configurations can play an important role in the heat transfer and mass transport during the different reactions of interest where MIEC membranes can be integrated [26,27,28,29].

### 2.2. Membrane Manufacture

An asymmetric all-La_0.6_Sr_0.4_Co_0.2_Fe_0.8_O_3–δ_ (LSCF) membrane was employed in this study. The LSCF membrane consisted of a dense layer of 22 ± 1.6 μm thickness and a support of 810–840 μm thickness with a porosity of 38 ± 1.6%. It was manufactured by tape casting, following the procedure described in a previous study [15]. A porous LSCF layer with a thickness of 13 ± 5 μm and a porosity of 37 ± 1 was also applied on the free surface of the dense membrane, aiming to improve the surface exchange kinetics of the membrane [3].

### 2.3. Membrane Sealing

Sealing of the LSCF membrane to the membrane module was made by reactive air brazing (RAB) using an Ag-based paste with a composition of 91.5 wt% Ag, 8 wt% CuO and 0.5 wt% Ti(H) (Innobraze GmbH, Esslingen am Neckar Germany). The paste was applied to the cavity of the membrane module by brushing, and the asymmetric membrane was placed and loaded with 20 g weight. It was heated to 750 °C (heating rate of 6 K/min), and then to 950 °C (heating rate of 10 K/min) where brazing took place for 1 h. Afterward, it was cooled to room temperature with a cooling rate of 100 K/h.

Before the sealing, the membrane module made of Inconel was annealed at 800 °C for 5 h in air to generate a Cr–oxide protective layer in order to prevent diffusion of the metal cations into the braze and evaporation.

### 2.4. Oxygen Permeation Measurements

Oxygen permeation studies were conducted in the metallic membrane reactor as described above. Argon was used as sweep gas on the dense membrane layer side (permeate side), and oxygen containing atmospheres were fed on the support side (feed side). The choice of this configuration was based on previous works on self-supported thin membranes [15,16]. The absolute pressure on the sweep side of the reactor was 2 bars, while it was 1.6 bars in the feed. A thermocouple was attached to the membrane in order to control the temperature. The O_2_ flux (J(O_2_)) was studied for various sweep gas flow rates and different oxygen partial pressures (*p*O_2_) in the feed. The O_2_ concentration in the permeate was analyzed by using a micro-GC Agilent 490 equipped with Molsieve 5A and Pora-Plot-Q glass capillary modules. Membrane gas leak-free conditions were ensured by continuously monitoring the nitrogen concentration in the sweep gas stream (permeate side). The detected N_2_ was always lower than 5% of the O_2_ detected in the sweep gas stream. Measurements were performed after 1 h of stabilization, and the GC analysis for each condition was repeated three times to minimize the analytical error. The standard deviation observed in the measurements ranged between 0.0005 and 0.014. Oxygen permeation was measured from 750 °C to 650 °C. The membrane was under operation for a total of 450 h. SEM and EDX analyses of the membrane cross-sections (as produced and after permeation measurements) were performed using a Zeiss Ultra 55 instrument (Zeiss, Jena, Germany). The samples were embedded at 300 mbar in a resin and subsequently polished to mirror finish.

## 3. Results

### 3.1. Oxygen Permeation Results

**Figure 3:**
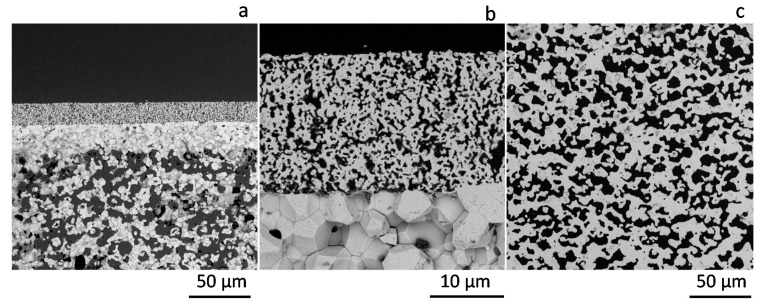
SEM micrographs of the cross-section of the membrane (**a**), catalytic layer (**b**) and support (**c**) after O_2_ permeation measurements.

Oxygen permeation was measured using an asymmetric LSCF membrane with a thickness of 22 ± 1.6 μm. A porous LSCF layer was coated on the dense side of the membrane in order to improve the catalytic activity of the surface and consequently the O_2_ permeation [4]. Micrographs of the cross section of the whole membrane and a detail of the porous catalytic layer and the support are shown in Figure 3. Sealing of the membrane to the metallic membrane module by RAB was successful, and no leaks between either side of the membrane were detected based on GC analysis of the effluent gases.

**Figure 4:**
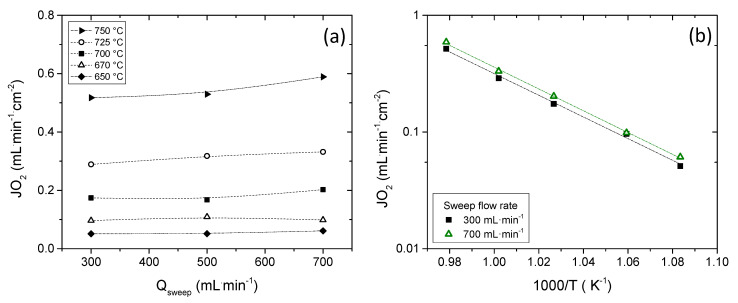
O_2_ flux as a function of the sweep flow rate (**a**) and as a function of the reciprocal temperature (**b**). Q_feed_ (synthetic air) = 300 mL·min^−1^.

An exhaustive study of the sweep flow rate and the feed side concentration influence on the O_2_ permeation was performed in co-current configuration. First, O_2_ permeation was measured between 750 °C and 650 °C by feeding 300 mL·min^−1^ of synthetic air at the support side and varying the sweep flow rate from 300 to 700 mL·min^−1^. O_2_ fluxes obtained as a function of the sweep flow rate at different temperatures are plotted in Figure 4a. The O_2_ flux remains practically constant with the increase in the sweep flow below 700 °C because no important gas diffusion resistances are expected due to the low O_2_ permeation flux. On the contrary, at temperatures above 700 °C, an increase in the O_2_ flux with the sweep flow is observed, this improvement being around 12% for 700 mL·min^−1^ argon sweep at 750 °C. The improvement is related to the dilution of oxygen in the permeate side. This gives rise to a higher oxygen driving force and an improvement of the mass transfer from the gas phase to the external surface of the porous activation layer [15,16]. The apparent activation energy (E_A_) remained almost constant regardless of the sweep flow rate, being 1.88 eV and 1.89 eV for 300 mL·min^−1^ and 700 mL·min^−1^, respectively, as observed in Figure 4b. This indicates that gas phase concentration polarization was not rate controlling. However, the activation energy is well above than what is reported for bulk diffusion in LSCF, i.e., 1.41 eV, which indicates a significant influence of the surface exchange kinetics. [33] For comparison, similar asymmetric LSCF membranes measured under analogous conditions [15] at temperatures between 600 °C and 700 °C showed an apparent activation energy of ~179 kJ/mol (1.86 eV) when no catalytic layer was applied, whereas this decreased to ~137 kJ/mol (1.42 eV) when an activation layer was coated. The E_A_ of 1.88 eV, thus, indicates important surface exchange limitations. Since the surface activation layer in our study does not change the activation energy, these surface exchange limitations do not result from the free membrane surface, but the low surface area at the membrane/support interface. These limitations can be alleviated by the improvement of the support and the catalytic layer by optimizing their structural parameters, especially the increase in the surface area and by infiltration of catalytic nanoparticles [19,20,34,35].

**Figure 5:**
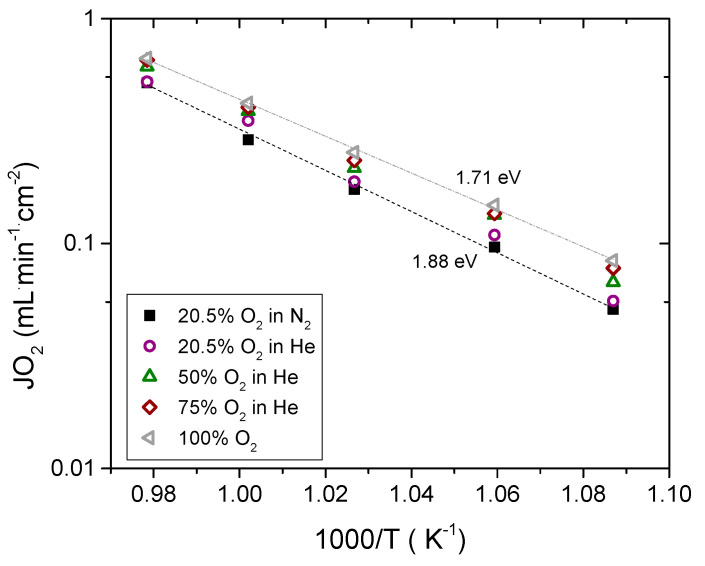
O_2_ permeation as a function of the reciprocal temperature and feed composition. Q_sweep_ = 300 mL·min^−1^ and Q_feed_ = 300 mL·min^−1^.

The influence of the O_2_ concentration in the feed was also evaluated. In addition, He was used instead of N_2_ for diluting the O_2_ in order to assess the polarization resistance of the porous support. The O_2_ flux variation by feeding different O_2_ concentrations as a function of the reciprocal temperature is plotted in Figure 5. The O_2_ flux increases with increasing *p*O_2_ due to the increase of the oxygen partial pressure difference between both sides of the membrane as it is postulated by the Wagner equation. However, at *p*O_2_ = 0.21 atm, the O_2_ flux increases when He is used instead of N_2_, ascribed to the faster diffusion of oxygen through helium in the porous substrate compared to N_2_ due to the higher gas diffusivity and lower viscosity [15].

Permeation follows an Arrhenius dependence, and the activation energies were 1.88 eV (189.99 kJ·mol^−1^) and 1.71 eV (164.87 kJ·mol^−1^) in synthetic air and pure O_2_, respectively. This decrease in the activation energy is attributed to the increase in the O_2_ concentration, improving the surface exchange coefficient k_ex_, which is typically proportional to *p*O_2_^0.5^ [15].

**Figure 6:**
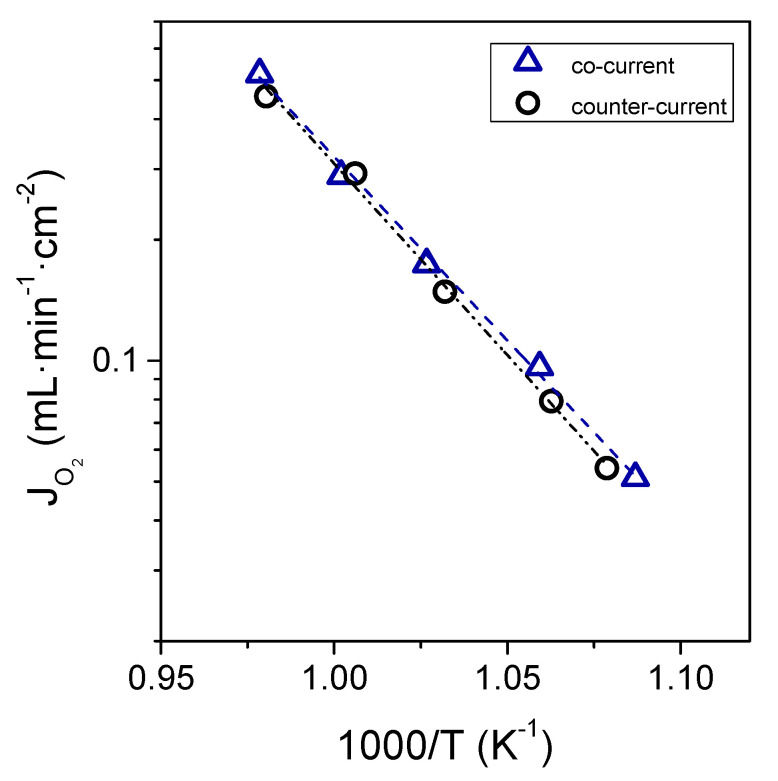
O_2_ permeation flux as a function of the reciprocal temperature for co-current and counter-current configuration. Q_sweep_ = 300 mL·min^−1^ and Q_feed_ (synthetic air) = 300 mL·min^−1^.

Depending on the reaction being conducted in the reactor, the flow mode, i.e., co- and counter-current configuration, can play an important role. In this work, O_2_ permeation was also measured in counter-current configuration. The O_2_ fluxes obtained in this configuration were practically the same as in co-current configuration and follow the same trend, as observed in Figure 6,where O_2_ permeation as a function of the reciprocal temperature is plotted (300 mL·min^−1^ of Ar and 300 mL·min^−1^ of synthetic air were used as sweep and feed streams, respectively).

These types of membranes and their integration in catalytic membrane reactors offer several advantages as compared with the conventional technology. The oxygen separation by using membrane technology is less energy intensive than conventional oxygen production, such as cryogenic distillation. In addition, catalytic membrane reactors offer lower unit/process volume, safe operation, minimization of secondary products (reducing the separation steps) and consequently, energy saving [3,9].

In fact, the coupling of oxygen selective membranes with high temperature reactions, such as oxidative coupling of methane and partial oxidation of methane, can offer different benefits when compared with conventional reactors (where O_2_ is co-fed). In these reactions, the distribution of oxygen allows to work at low oxygen concentration in the reactor, maximizing the products yield, in addition to operating below the explosive limits for CH_4_/O_2_ mixtures [9,36,37]. Furthermore, two different reactions could be performed in the same membrane. An example of this coupling of processes was reported by Jiang et. al. where water splitting and partial oxidation of methane were performed simultaneously by using a MIEC hollow fiber [38].

### 3.2. Membrane and Sealing Characterization after Permeation Measurements

The membrane was characterized by SEM after 450 h on stream and several thermal cycles. After the permeation measurements shown in the previous section, membrane leak increased and consequently the measurement was stopped.

**Figure 7:**
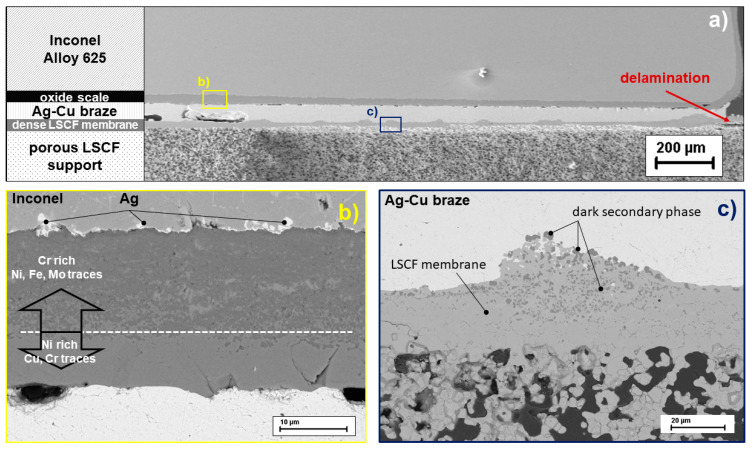
SEM image of the brazing area Inconel Alloy 625/Ag-Cu/LSCF membrane: overview (**a**), interface Inconel alloy—Ag-Cu braze (**b**), and interface Ag-Cu braze—LSCF Membrane (**c**).

The analysis of the joining was conducted on polished cross-sections using SEM and EDX. An overview shows the metallic support made of Inconel, the oxide protective layer, the Ag-Cu braze, the dense LSCF membrane layer and the porous LSCF support (Figure 7a). The good adhesion of the Ag-Cu braze to the oxide protective layer and the dense LSCF membrane is evident. However, microstructural changes in the protective and membrane layer are visible in detailed images compared to non-exposed joints. Along the metal surface (Inconel), a Cr-oxide protective layer was created in order to prevent diffusion of the metal cations into the braze and evaporation. The BSE image of this protective layer is shown in Figure 7b, where two phases are identified. The darker phase, which is in contact with the metallic support, contains mainly Cr with small traces of Ni, Fe, and Mo, as expected for the protective layer. Traces of Ag are found at the interface that could have been introduced by the polishing process. The light phase of the protective layer, which is in contact with the Ag-Cu solder, has Ni as main element and contains Cu and only traces of Cr. It can be seen that the Cr-oxide protective layer has not completely stopped the diffusion of metal cations toward the Ag-Cu solder.

The Ag-Cu solder joint appears free of foreign phases. However, the contact surface between the solder and the dense LSCF membrane shows bulge-like unevenness (Figure 7c) compared to the previously smooth membrane surface.

**Figure 8:**
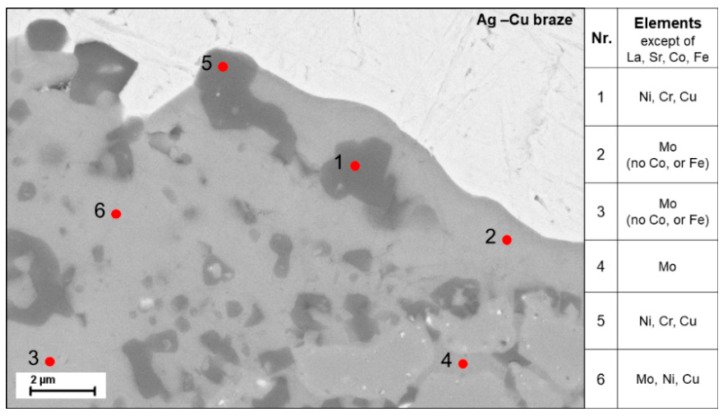
BSE image of the interface Ag-Cu braze—LSCF membrane with EDX results shown in the table.

The BSE detail image of a bulge shows a dark contaminant phase in the lighter LSCF matrix (Figure 8) The grain structure is already clearly dissolved. It is also visible that the grain boundaries below the bulge are decorated with a dark contaminant phase. The EDX examination shows that the dark phase (pointed as 1 and 5) consists mainly of Ni, Cr, Cu and the elements of LSCF, while in the light grey phase (pointed as 2 and 3) Mo is the main element detected while La and Sr are present, but neither Co nor Fe. Point 4 shows a light grey grain boundary decoration, which consists mainly of Mo. The diffusion of the metal cations, mainly Mo, from the metallic support into the LSCF seems to change the structure of the membrane layer. Possibly, the Mo cations lead to a change in chemical composition of the LSCF, which could explain the dissolution of the LSCF grain structure and the compositions in point 2 and 3.

**Figure 9:**
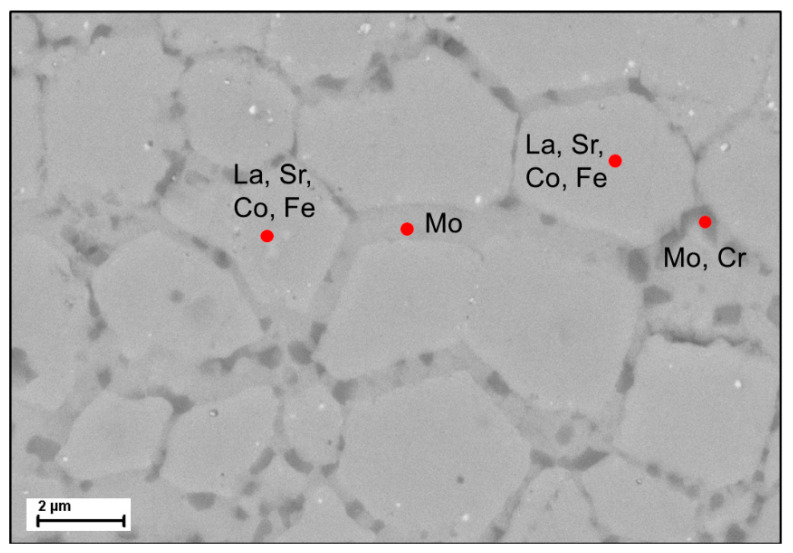
BSE image of the dense membrane layer with EDX results.

Furthermore, the diffusion leads to an increase in volume in the membrane layer, which induces compressive stresses. Those are compensated by the solder in the joining area. Directly behind the joint, however, delamination of such a bulge occurs (see Figure 9). The crack does not run along the interface between the dense and porous layer, as expected, but within the membrane. Here, too, the grain boundaries are occupied by the Mo-rich second phase. Therefore, this phase seems to have a negative influence on the mechanical strength.

In summary, Ni, Cr and Mo can pass through the Cr-oxide protective layer of the Inconel support. Mo seems to pass through the solder and damages the LSCF ceramic. Here, the role of the Cu contained in the solder has not yet been considered. A detailed investigation of the damage mechanisms will be the subject of further work.

## 4. Conclusions

The integration of MIEC ceramic membranes in compact metallic reactors allows the operation of these types of membranes at high pressures and present the added advantage of an enhanced mass transport and heat transfer. In addition, compact metallic reactors can be equipped with microchannels allowing the rapid extraction of the products and providing a high membrane surface area to catalyst volume ratio.

Because of these interesting features, a compact metallic reactor for the integration of MIEC ceramic membranes was constructed. The reactor consisted of a housing made of Nicrofer^®^ 3220H/Alloy 800 with two separated parts provided of inlets and outlets for the permeating and feed chamber. A membrane module that separates both chambers of the reactor was developed to allocate the MIEC ceramic membrane.

In this study, the viability of the MIEC ceramic membranes integration in the developed compact metallic reactor was demonstrated. For this purpose, O_2_ permeation studies were conducted using an asymmetric La_0.6_Sr_0.4_Co_0.2_Fe_0.8_O_3–δ_ membrane. The membrane was leak-tight integrated in the module by reactive air brazing (RAB) using Ag-based paste. The influence of the temperature, sweep flow rates and oxygen partial pressure in the feed on the O_2_ permeation flux was evaluated.

No important gas diffusion resistances were observed in the studied conditions. O_2_ permeation was mainly controlled by the surface exchange kinetics despite the applied catalytic layer on the dense membrane. The low surface exchange kinetics are attributed to the low specific surface area of the support in the proximity of the dense membrane layer. In order to improve the surface exchange kinetics, the porous support and the catalytic layer can be optimized by tuning the structural parameters, such as specific surface area, porosity, tortuosity and average pore opening diameter. In addition, the infiltration of catalytic nanoparticles can boost the surface exchange kinetics.

The MIEC membrane was continuously operating during 450 h without any detectable leak and performance degradation. However, after this time, a leak was clearly observed. The characterization of the membrane-metal joint by SEM evidenced a good adhesion of the Ag-Cu braze to the oxide protective layer of the steel and the dense LSCF membrane layer. However, microstructural changes in the protective and membrane layers were detected. The Cr-oxide protective layer did not totally hinder the diffusion of metal cations toward the Ag-CuO solder and the contact surface between the solder. In order to avoid the diffusion of cations that hinders the long-term operation of the membrane, more studies are needed. These studies should be based on the use of alternative metallic alloys, protective layers or the development of thin membranes on porous metallic supports.

In summary, this work represents a proof of concept of the integration of ceramic membranes in compact metallic reactors that are promising for the development of modular catalytic membrane reactors.

## Data Availability

Not applicable.
